# Predicting women with depressive symptoms postpartum with machine learning methods

**DOI:** 10.1038/s41598-021-86368-y

**Published:** 2021-04-12

**Authors:** Sam Andersson, Deepti R. Bathula, Stavros I. Iliadis, Martin Walter, Alkistis Skalkidou

**Affiliations:** 1 Department of Women’s and Children’s Health, Uppsala University, 751 85 Uppsala, Sweden; 2 Department of Computer Science and Engineering, Indian Institute of Technology Ropar, Rupnagar, Punjab 140001 India; 3 Department of Psychiatry and Psychotherapy, University Hospital Jena, Jena, Germany; 4 Department of Psychiatry and Psychotherapy, Eberhardt Karls University, Tübingen, Germany; 5 Department of Behavioral Neurology, Leibniz Institute for Neurobiology, Magdeburg, Germany

**Keywords:** Machine learning, Risk factors, Depression

## Abstract

Postpartum depression (PPD) is a detrimental health condition that affects 12% of new mothers. Despite negative effects on mothers’ and children’s health, many women do not receive adequate care. Preventive interventions are cost-efficient among high-risk women, but our ability to identify these is poor. We leveraged the power of clinical, demographic, and psychometric data to assess if machine learning methods can make accurate predictions of postpartum depression. Data were obtained from a population-based prospective cohort study in Uppsala, Sweden, collected between 2009 and 2018 (BASIC study, n = 4313). Sub-analyses among women without previous depression were performed. The extremely randomized trees method provided robust performance with highest accuracy and well-balanced sensitivity and specificity (accuracy 73%, sensitivity 72%, specificity 75%, positive predictive value 33%, negative predictive value 94%, area under the curve 81%). Among women without earlier mental health issues, the accuracy was 64%. The variables setting women at most risk for PPD were depression and anxiety during pregnancy, as well as variables related to resilience and personality. Future clinical models that could be implemented directly after delivery might consider including these variables in order to identify women at high risk for postpartum depression to facilitate individualized follow-up and cost-effectiveness.

## Introduction

Postpartum depression (PPD), defined as having an episode of minor or major depression during pregnancy or up to one year after giving birth, is a relatively common condition that affects 8–15% of new mothers in Sweden every year^1,2^. The etiology of PPD is not well understood, but the condition likely arises from a combination of psychological, psychosocial and biological factors^3,4^. The most well documented biological risk factors for PPD are hypothalamic–pituitary–adrenal axis dysregulation, inflammatory processes, genetic vulnerability, and allopregnanolone withdrawal^4^. The strongest psychosocial factors are previous depression, severe life events, some forms of chronic stress and relationship struggles^4,5^. The role of resilience and personality have been lately also gaining attention^6,7^.

PPD is a condition that can have devastating effects on the mothers, as well as their children^8,9^. Mothers may experience persistent doubts about their ability to care for the child, have difficulties bonding with their child, and also have thoughts about hurting the child^2^. Moreover, PPD can affect a child’s development by interfering with the mother-infant relationship^10,11^. For instance, children of mothers with PPD have greater cognitive, behavioral and interpersonal problems compared to children of mothers without PPD^12,13^. Despite PPD being a detrimental health condition for many women, numerous affected women fail to receive adequate care^14^. There exist several effective treatments and interventions for PPD^14–16^, but they are only cost-effective among high-risk women. The idea of prenatal prediction of PPD has existed for several years and early studies using more traditional methods attempted to predict women at risk by prenatal assessment of critical variables^17^. However, to date, there has been no effective way to predict women at risk for the development of depressive symptoms postpartum.

Traditional statistical methods allow researchers to estimate risks by sequentially analysing the associations mainly between two variables, often controlling for the effect of others. Further, machine learning (ML) methods enable researchers to iteratively and simultaneously analyse multiple interacting associations between variables^18^ as well as to devise data-driven predictive models that then can be evaluated by quantifying the performance metrics across all models in order to find the best predictive model. The power of ML allows for the analysis of complex non-linear relationships and even the integration and pooling of multiple different data-types from several sources^19–21^. Over the last decade, there has been a steady increase in the use of ML in medicine and its effects can be observed in many fields including oncology^22–25^, cardiology and hematology^26,27^, critical care^28,29^, and psychiatry^30–35^. Importantly, PPD represents a unique case in which a moderately high chance to develop a serious psychiatric condition is coupled with a very precise temporal prediction of when such symptoms are to be expected. As such, and considering PPDs substantial societal burden, ML-based risk classification can be applied in an ideal situation with high expected societal benefit. With approximately 120,000 annual births in Sweden and the typical prevalence of PPD at 12% among women who nearly in their entirety present with a multitude of adaptations after childbirth, close monitoring of the whole population for early depressive sentinels after childbirth seems hardly feasible in reality. In contrast, close follow-up among high risk groups during midwife or nurse-led postpartum assessments may strongly contribute to more tailored and cost-efficient maternal perinatal mental care services.

However, despite promising results in other fields, relatively few studies have been performed using ML in the field of perinatal mental health. An early study in the field could predict PPD with an accuracy of 84% by use of multilayer perceptrons and assessment of 16 variables^36^. A recent pilot study used ML algorithms applied to data extracted from electronic health records to show that ML models can be utilized to predict PPD and identify critical variables that conform with known risk variables such as race, demographics, threatened abortion, prenatal mental disorder, anxiety, and an earlier episode of major depression^34^. Another study also developed models to predict PPD, which were then integrated into a mobile application platform to be used by pregnant women^37^, while a recently published study compared four PPD prediction models that comprised demographic, social and mental health data^38^. In the latter study, psychological resilience was pointed out as an important predictive factor. However, these studies have been limited by either sample size or richness of data. Finally, in a recently published study, Zhang et al. proposed a machine learning based framework for PPD risk prediction in pregnancy, using electronic health record data^39^.

To date, our study is the first using a population-based, large and rich dataset, including a wide range of clinical and psychometric self-report and medical journal-derived variables and evaluating a range of different ML algorithms against each other, and also after stratification for earlier or pregnancy depression, to provide a robust screening tool, at discharge from the delivery ward, for predicting women at risk for developing depressive symptoms later in the postpartum period.

Hence, we aim to predict women at risk for depressive symptoms at 6 weeks postpartum, from clinical, demographic, and psychometric questionnaire data available after childbirth, by use of machine learning methods.

## Results

### Descriptive statistics

Table 1 shows summary statistics of the study population by depressive symptom status at 6 weeks postpartum. Results are presented as frequencies and relative frequencies within EPDS status [N (%)] or median (interquartile range) for sociodemographic, clinical and questionnaire variables. Of the 4313 participants in the study, 577 had depressive symptoms at 6 weeks postpartum. The mean age for both groups was 31 years. Differences were seen among women with depressive symptoms and women without depressive symptoms across sociodemographic variables like education, employment, and country of origin, as well as many other variables known as risk factors for postpartum depression. A greater proportion of women with depressive symptoms postpartum did not receive adequate support from their partner and were not breastfeeding.

**Table 1:** Characteristics of the study participants by depression status at 6 weeks postpartum (EPDS^a^ score 0–11 vs. 12–30) (*n* = 4313).

Characteristics	Missing values	EPDS^a^ at 6 weeks postpartum		p^o^
		0–11 (*n* = 3736)	12–30 (*n* = 577)	
**N (%) or median (IQR**^**b**^**)**				
Age (years)	3	31 (6)	31 (6)	0.247
BMI (kg/m^2^) before pregnancy	162	22.8 (4.4)	23.3 (5.5)	**0.001**
Height at gestational week 17 (cm)	151	168 (9)	167 (8)	0.305
University level education (vs. less)	268	2734 (78%)	387 (72%)	**0.001**
Employment	264			**< 0.001**
Full-time work		2291 (65%)	310 (57%)	
Part-time work		696 (20%)	102 (19%)	
Studying		242 (7%)	49 (9%)	
Maternity leave		110 (3%)	15 (3%)	
Sick-leave		89 (3%)	26 (5%)	
Unemployed		78 (2%)	41 (7%)	
Marital status (single vs. married/cohabiting*)*	9	55 (1.5%)	16 (2.8%)	**0.022**
Country of origin (scandinavia vs. other)	184	3311 (93%)	499 (91%)	0.193
Depression history^c^	185	959 (27%)	296 (54%)	**< 0.001**
Contact with psychiatrist (self-reported)^d^	250	285 (8%)	98 (18%)	**< 0.001**
Contact with psychologist (self-reported)^d^	250	1190 (34%)	299 (55%)	**< 0.001**
Hypomanic episodes (1 or more vs. none)^d^	270	141 (4.0%)	50 (9.3%)	**< 0.001**
Premenstrual syndrome history (based on the ICD and ACOG criteria)^d^	303	169 (4.9%)	79 (15%)	**< 0.001**
History of pregnancy loss	2072	21 (1.1%)	5 (1.6%)	0.477
Parity	303			0.072
0		1934 (55%)	309 (60%)	
1		1082 (31%)	154 (30%)	
2 or more		477 (14%)	54 (10%)	
Smoking, ever	175	1157 (32%)	197 (36%)	0.096
Snuff, before pregnancy	402	251 (7.4%)	40 (7.6%)	0.846
Sleep before pregnancy (hours per day)	179			**0.005**
< 6		124 (3%)	32 (6%)	
6–8		29 27 (82%)	421 (77%)	
> 8		534 (15%)	96 (17%)	
Migraine^d^	295	584 (17%)	120 (22%)	**0.002**
Irritable bowel syndrome^d^	295	127 (3.7%)	32 (5.9%)	**0.011**
Alcohol problems^d^	295	5 (0.1%)	5 (0.9%)	**0.001**
Allergies^d^	295	764 (22%)	119 (22%)	0.951
Endocrine problems^d^	295	124 (3.6%)	25 (4.6%)	0.220
Hypertension^d^	295	55 (1.6%)	14 (2.6%)	0.091
Pain problems^d^	295	137 (3.9%)	43 (8.0%)	**< 0.001**
Intimate partner violence^d^	275	362 (10%)	107 (20%)	**< 0.001**
**Pregnancy-related variables**				
Planned pregnancy	288	481 (14%)	121 (22%)	**< 0.001**
Assisted reproductive technology treatment	424	369 (11%)	46 (8.7%)	0.120
Several ultrasounds during gestation	298	2241 (65%)	371 (69%)	**0.048**
Fear of childbirth	254			**< 0.001**
No fear		2774 (79%)	336 (62%)	
Fear of caesarean section		339 (9.6%)	73 (14%)	
Fear of vaginal delivery		208 (5.8%)	61 (11%)	
Severe fear		200 (5.6%)	68 (13%)	
Visit at fear of childbirth support unit	250	77 (2.2%)	19 (3.5%)	**0.061**
Negative delivery expectations	305	504 (15%)	131 (25%)	**< 0.001**
Pregnancy nausea	281			**< 0.001**
No		705 (20%)	74 (14%)	
Yes, without medication		2313 (66%)	363 (67%)	
Yes, with medication		476 (14%)	101 (19%)	
SSRI^f^ use during pregnancy	368	132 (3.9%)	39 (7.5%)	**0.001**
Anxiety during pregnancy^g^	237	1032 (29%)	387 (72%)	**< 0.001**
Depression during pregnancy^h^	162	472 (13%)	298 (53%)	**< 0.001**
Sleep during pregnancy (hours per day)^i^	266			**< 0.001**
< 6		322 (9%)	98 (18%)	
6–8		2340 (67%)	332 (61%)	
> 8		843 (24%)	112 (21%)	
Self-reported pregnancy complications (any)^j^	354	1471 (42.7%)	215 (42.0%)	0.771
Gestational diabetes^j^	136	35 (1.0%)	19 (3.5%)	**< 0.001**
Preeclampsia^j^	136	117 (3.2%)	23 (4.2%)	0.242
Anaemia^j^	354	103 (3.0%)	21 (4.1%)	0.177
Hypertension^j^	347	165 (4.8%)	41 (8.0%)	**0.002**
Symphysiolysis^j^	683	1212 (39%)	228 (47%)	**< 0.001**
Pregnancy length (days)	194	280 (13)	280 (14)	0.103
**Childbirth-related variables**				
Delivery month	1	6 (6)	6 (6)	0.386
Induction	301	651 (18.6%)	100 (19.3%)	0.697
Mode of delivery	0			0.107
Spontaneous vaginal		2795 (75%)	402 (70%)	
Elective caesarean section		271 (7%)	47 (8%)	
Acute caesarean section		320 (9%)	64 (11%)	
Emergency caesarean section		33 (1%)	7 (1%)	
Instrumental delivery		317 (8%)	57 (10%)	
Postpartum hemorrhage (≥ 1000 ml vs. < 1000 ml)	301	221 (6.3%)	46 (8.9%)	**0.028**
Epidural anaesthesia	286	1306 (37%)	238 (46%)	**< 0.001**
Laceration (Grade III/IV vs. I/II)	146	103 (2.8%)	14 (2.6%)	0.712
**Infant-related variables**				
Gender (female)	171	1733 (48%)	269 (50%)	0.458
Birthweight (kg)	204	3.6 (0.65)	3.6 (0.70)	0.201
Birth length (cm)	219	51 (2.5)	51 (3.0)	0.242
Head circumference (cm)	1184	35 (2.0)	35 (2.0)	0.672
Apgar score (1st min)	222			**0.027**
0–3		26 (1%)	6 (1%)	
4–6		111 (3%)	28 (5%)	
7–10		3418 (96%)	502 (94%)	
Umbilical artery base deficit	991	− 2.9 (4.2)	− 2.6 (4.6)	0.250
Umbilical artery pH	949	7.3 (0.10)	7.3 (0.11)	0.519
Newborn admission to neonatal unit	286	300 (8.6%)	69 (13%)	**< 0.001**
**Postpartum variables**				
Negative delivery experience	411	217 (6.4%)	85 (17%)	**< 0.001**
Partner helpful with infant	40			**< 0.001**
Yes, a lot		2334 (63%)	300 (53%)	
Yes, a little		1281 (35%)	235 (41%)	
No		87 (2%)	36 (6%)	
Breastfeeding	11			**< 0.001**
Yes, exclusive		2918 (78%)	354 (62%)	
Yes, non-exclusive		576 (16%)	146 (25%)	
No		233 (6%)	75 (13%)	
Stressful life events past 6 months (3–30 vs. 0–2)	13	395 (11%)	129 (22%)	**< 0.001**
**Psychometric scales**				
ASQ [pregnancy week 32]^k^				
Distance	2658	2.8 (1.1)	3.4 (1.4)	**< 0.001**
Insecure and repudiation connection	2658	2.1 (0.86)	2.3 (1.0)	**< 0.001**
Trust	2658	4.8 (1.0)	4.3 (1.3)	**< 0.001**
Consents	2658	2.9 (1.3)	3.7 (1.4)	**< 0.001**
Relation	2658	3.0 (1.0)	3.5 (1.1)	**< 0.001**
SOC (total score) [pregnancy week 32]^l^	2095	154 (28)	131 (31)	**< 0.001**
LITE (number of events) [12 months postpartum]^m^	1696	3.0 (3.0)	4.0 (4.0)	**< 0.001**
Resilience Scale-14 [pregnancy week 32]	1982	81 (15)	72 (24)	**< 0.001**
Beck anxiety inventory (moderate/severe vs. minimal/mild) [pregnancy week 32]	2015	299 (15%)	162 (50%)	**< 0.001**
SSP-Neuroticism score [pregnancy week 32]^n^	3230	328 (66)	387 (64)	**< 0.001**
SSP-Aggressiveness score [pregnancy week 32]	3236	195 (26)	203 (26)	**0.001**
SSP-Sensation Seeking score [pregnancy week 32]	3224	94 (19)	95 (17)	0.258

Bold values indicate *p* < 0.05.

^a^Edinburgh Postnatal Depression Scale.

^b^Interquartile range.

^c^Self-reported and/or diagnosed by psychiatrist.

^d^Pregnancy week 17, self-reported.

^e^EPDS ≥ 12 at pregnancy week 17 or 32 or at delivery.

^f^Selective serotonin reuptake inhibitors.

^g^Based on either the Beck Anxiety Inventory, the State Trait Anxiety Inventory (STAI) or the anxiety subscale of the EPDS (EPDS 3A, items 3–5).

^h^EPDS ≥ 12 at pregnancy week 17, 32 or 38.

^i^Pregnancy week 32.

^j^Self-reported, pregnancy week 32.

^k^Attachment Style Questionnaire.

^l^Sense of Coherence Questionnaire-29.

^m^Lifetime Instances of Traumatic Events.

^n^Swedish Universities scales of Personality.

^o^Based on the Mann–Whitney test for continuous variables and the Pearson Chi-square test for the categorical variables.

### Classification graphs

**Figure 1:**
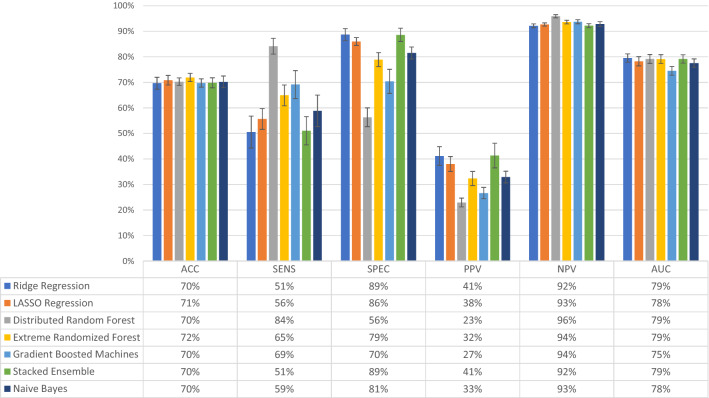
Evaluation of model performance in the dataset containing only background, medical and pregnancy-related variables (n = 4277 women). The models tested were Ridge Regression, LASSO Regression, Distributed Random Forest, Extremely Randomized Trees, Gradient Boosted Machines, Stacked Ensemble, and Naïve Bayes. Models were assessed for accuracy (ACC), sensitivity (SENS), specificity (SPEC), positive predictive value (PPV), negative predictive value (NPV), and area under the curve (AUC), the outcome being depressive symptoms at 6 weeks postpartum. The bars represent the level of performance measures (in percent) and the table below the bar plot presents the exact numerical values. Error bars represent one standard deviation from the mean.

To evaluate whether ML can predict women with depressive symptoms, two datasets were used, namely the BP variables and the combined dataset, that includes the BP variables and three psychometric questionnaires (RS, SOC, and VPSQ). Performance of different ML models was first evaluated for the BP data (Fig. 1). The performance metrics for Ridge Regression, LASSO Regression, Gradient Boosting Machines, Distributed Radom Forests (DRF), Extreme Randomized Forests (XRT), Naïve Bayes and Stacked Ensembles models are shown. Balanced accuracy, NPV and AUC were quite similar across the models, with accuracy reaching 72% and AUC 79% for XRT. NPV was over 92% for all models. Sensitivity was quite low and together with specificity and PPV, they varied between the models. Sensitivity was highest for DRF at 84%, while only 65% for XRT; DRF had though the lowest specificity and PPV. The highest PPV was observed for Ridge Regression and Stacked Ensemble, at 41%.

**Figure 2:**
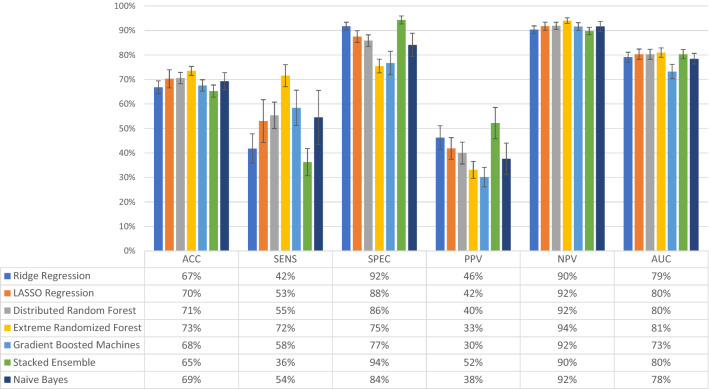
Evaluation of model performance in the total combined dataset (n = 2385 women). The combined dataset contained the background, medical and pregnancy-related variables, as well as answers to the questionnaires Resilience-14, Sense of Coherence-29 and Vulnerable Personality Scale Questionnaire. The models tested were Ridge Regression, LASSO Regression, Distributed Random Forest, Extremely Randomized Trees, Gradient Boosted Machines, Stacked Ensemble, and Naïve Bayes. Models were assessed for accuracy (ACC), sensitivity (SENS), specificity (SPEC), positive predictive value (PPV), negative predictive value (NPV), and area under the curve (AUC), the outcome being depressive symptoms at 6 weeks postpartum. The bars represent the level of performance measures (in percent) and the table below the bar plot presents the exact numerical values. Error bars represent one standard deviation from the mean.

Performance of different ML models was then evaluated for the combined dataset, even including psychometric measures (Fig. 2). Performance metrics for the same models showed that NPV was still over 90% for all models, but otherwise, similar levels of accuracy and AUC were observed. More variability among the models was observed for sensitivity, specificity and PPV. XTR had the highest accuracy (at 73%) and AUC (at 81%) among all models, with a balance in sensitivity at 72% and specificity at 75%; PPV was at 33% and NPV at 94%. As this balancing act is an essential attribute of predictive models based on imbalanced datasets the subsequent experimental analysis was provided using only XRT.

**Figure 3:**
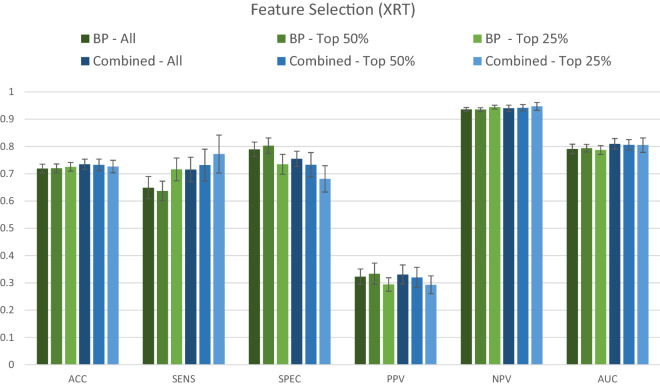
Comparative performance of the dataset containing only background, medical history and pregnancy-related variables (BP) and the combined dataset (BP + RS + SOC + VPSQ). The Extremely Randomized Trees (XRT) algorithm was used to compare the performance of the two datasets for predicting depression at 6 weeks postpartum. Models were assessed for accuracy (ACC), sensitivity (SENS), specificity (SPEC), positive predictive value (PPV), negative predictive value (NPV), and area under the curve (AUC). The variable selection procedure shows results when All (100%), Top 50%, and Top 25% of variables were retained, ranked according to Mean Decrease in Impurity (MDI) relevance.

Comparative performance of the XRT model using all variables, the top 50%, and the top 25% variables, for both the BP and the combined dataset is shown in Fig. 3. There was an apparent trade-off between model sensitivity and specificity, which were both affected by dataset used and percent of variables included (Fig. 3). Sensitivity was highest with use of only 25% of the combined dataset, while specificity was highest with the use of the top 50% of the BP dataset. None among the other measures were greatly affected by either dataset used or percent of variables included (a trend to lower PPV when 25% of variables used was noted). The AUC curves corresponding to Figs. 2 and 3 are available in the supplementary material (Supplementary Figure 1).

**Figure 4:**
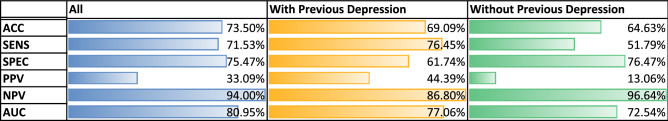
Stratified classification graphs for Extreme Randomized Forest (XRT) model, by pregnancy/previous depression status. Results presented for all women (All, n = 2385, of which 14% had postpartum depression, PPD), women with depression during current pregnancy or earlier in life (With Previous Depression, n = 971, of which 27% had PPD), and women without any previous depression episode (Without Previous Depression, n = 1414, of which 6% had PPD). For each category, models were assessed for accuracy (ACC), sensitivity (SENS), specificity (SPEC), positive predictive value (PPV), negative predictive value (NPV), and area under the curve (AUC).

The results for the performance of the XRT models after stratification for previous depression are shown in Fig. 4. For all women, XRT achieved a balanced accuracy of 73%, a sensitivity of 72%, a specificity of 75%, a positive predictive value of 33%, a negative predictive value of 94% and an AUC of 81%. For women with depression in pregnancy or earlier in life, XRT achieved a balanced accuracy of 69%, a sensitivity of 76%, a specificity of 61%, a positive predictive value of 44%, a negative predictive value of 87% and an AUC of 77%. For women without any previous depressive episode, balanced accuracy was 64%, sensitivity 52%, specificity 76%, positive predictive value of 13%, negative predictive value 97% and AUC of 73% (Fig. 4). Among the results from analyses of the individual questionnaires, no single one achieved an accuracy of more than 70% (Supplementary Figure 2).

### Variable importance

**Figure 5:**
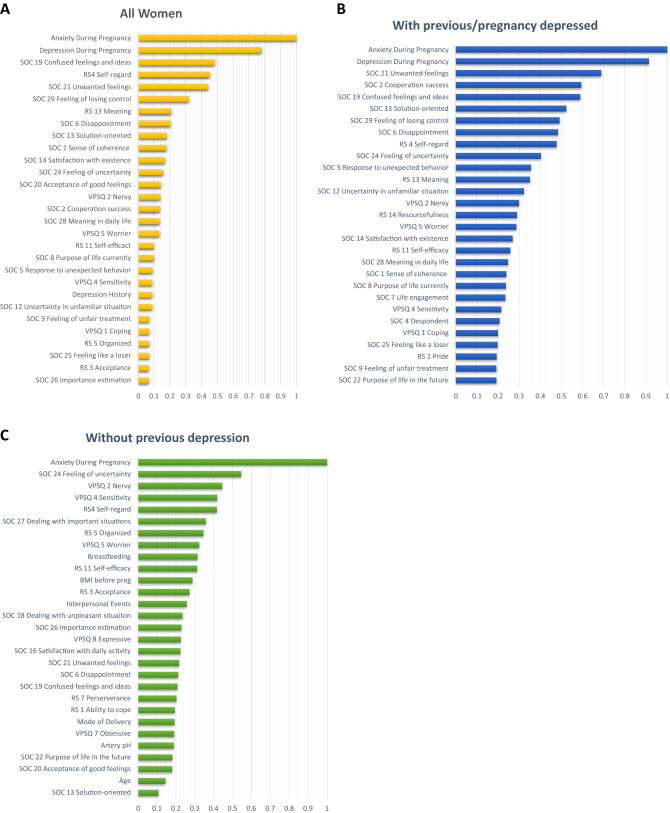
Ranked importance of the assessed variables using the Extremely Randomized Trees (XRT) models in the combined dataset, considering the women with different previous depression status. Results presented for all women (**A**), All women (n = 2385), (**B**) women with depression during current pregnancy or earlier in life (Previous/pregnancy depression, n = 971), and (**C**) women without any previous depression episode (No previous depression, n = 1414). The graphs depict the variable importance as a relative measure that is scaled to a maximum of 1.0. The x-axis represents the relative contribution to the classification algorithm of the corresponding feature on the y-axis.

The 25 most important variables by MDI based on Distributed Random Forests (DRF) models, considering the women with different previous depression status are shown in Fig. 5. For all women, Anxiety During Pregnancy and Depressive During Pregnancy stand out as the two most important variables (importance level above 0.7) (Fig. 5A). The variables following in importance were questions included in the psychometric instruments, except for history of depression. Similarly, for women with previous depression, Anxiety During Pregnancy and Depressive During Pregnancy stand out as important variables for the presence of depression postpartum (importance level above 0.9) (Fig. 5B). Finally, for women without depression, Anxiety During Pregnancy was the absolutely most important variable (importance level of 1) (Fig. 5C). Even here, variables relating to resilience, sense of coherence and personality followed, but interestingly, variables such as breastfeeding, BMI, traumatic events in childhood, mode of delivery, hypoxia in the newborn and age place among the top 25 variables.

**Figure 6:**
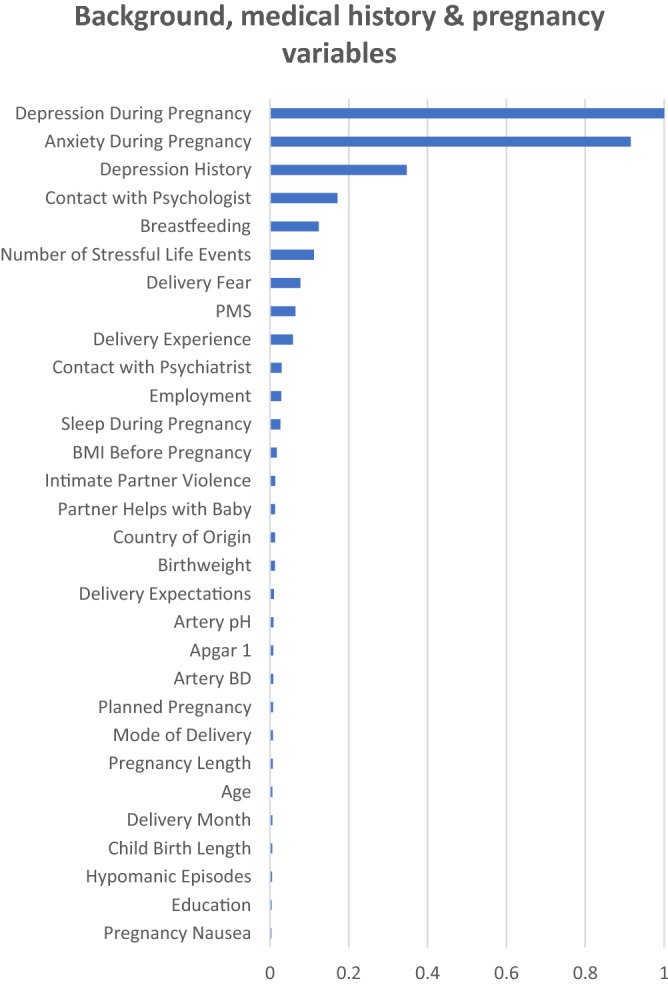
Ranked importance of the assessed background, medical history and pregnancy variables for all women (n = 4277) using Extremely Randomized Trees (XRT) models. The top 25% of the variables are reported**.** The x-axis represents the relative contribution of the corresponding variable to the classification algorithm.

The 25 most important variables based only on BP variables for all women (n = 4313) can be found in Fig. 6. The two variables that have an importance level above 0.9 are again Depression During Pregnancy and Anxiety During Pregnancy. The next variable with an importance level above 0.3 is Depression History, while the remaining rate below 0.2.

Including only the top 20 variables, the AUC is only reduced by 1% to 0.79, including just 10 variables reduced the AUC by 2% to ~ 0.78, while after including just 5 variables reduced the AUC by 3% to ~ 0.77. For the previously non-depressed group, including 10 variables gives an AUC of 0.72, and 5 variables an AUC of 0.71.

## Discussion

In this study, we evaluated a range of different machine learning (ML) methods to predict pregnant women at risk for postpartum depressive (PPD) symptoms. The classification performance of the chosen ML algorithms was not significantly different in regard to accuracy, NPV, AUC measures. However, variations were more pronounced in regard to sensitivity, specificity and PPV. In general, as expected, an inverse relationship is observed in performance with respect to sensitivity and specificity. Furthermore, PPV is considerably lower than NPV due to low prevalence of PPD, as expected.

Overall, XRT provides robust performance with highest accuracy and well-balanced sensitivity and specificity. Addition of resilience and personality self-reported variables to the background, medical history and pregnancy-related variables provides marginal improvement in both accuracy and AUC. It is nevertheless of note that these extra variables boost the sensitivity of the XRT model substantially for only a slight drop in specificity. As this does not depend on the lower sample size used for the second step of analyses involving personality and resilience measures, it could be hypothesized that there is either a certain redundancy between variables, e.g. that low resilience is a core feature among depressed patients during pregnancy, or that anxiety and depression measures, available for all patients, have such a strong predictive value that the further addition of variables does not greatly improve accuracy.

These results suggest a possible benefit of using ML to screen new mothers at discharge from the delivery ward in order to identify those at high risk for postpartum depressive symptoms. However, because of the low PPV across all models, due to the relatively low prevalence of PPD at 12%, one would expect that many women identified at high risk would in the end not get depressed. On the other hand, these methods may nevertheless permit the identification of a high-risk group, to which preventive interventions would be offered in a cost-effective way, mainly by avoiding large costs related to full-blown depressive episodes postpartum. These could include the provision of extra support as well as more focused and longitudinal assessments in these mothers. Furthermore, the variables included in the BASIC study refer to easily acquired web-based self-reports, which support their use for screening purposes. Because of the high NPV, we would not expect many women not identified as high risk to develop depression postpartum. As such, the application of our classification algorithms would boost cost-effectiveness, allowing for a tailored resource allocation towards the mothers initially identified at risk versus a more widespread follow up of all mothers; in the low-risk group, assessments could be limited to single timepoints, as is praxis today. As PPD affects more than 16,000 families every year in Sweden alone, with high associated costs, estimated at $30,000 per mother-infant pair for untreated peripartum mood disorders, preventive efforts would have substantial societal benefits^40^.

It is interesting that performance metrics, especially accuracy and AUC, remain stable even when the number of variables used in the models is reduced from 100 to 50% and even to 25% of all variables available, and AUC is relatively stable even at 5–10 variables. As discussed above, this is in line with the thought that there is some redundancy when it comes to the variables included, with depression and anxiety during pregnancy being highly correlated with some background and medical history variables, and possibly mediating their association with PPD. It is thus intriguing to observe that only among non-previously depressed, variables such as breastfeeding, BMI, traumatic interpersonal events in childhood, mode of delivery, infant hypoxia and age are emerging as important for prediction, along with resilience and personality variables, which are otherwise more prominent among those earlier depressed. This is important to have in mind when developing screening strategies; the variables used might need to be adjusted for the group of women with previous depression. Anxiety during pregnancy continues to be very predictive in both groups. The stability of the performance measures however, indicates that an abbreviated survey can be used to screen without significantly affecting predictive power.

Among possible explanations for the somewhat lower accuracy in both the depressed group (earlier or during pregnancy) (n = 971, accuracy = 69%) and never-depressed subgroups (n = 1414, accuracy = 64%) are the lower sample sizes as well as a relatively decreased variability in the data (the algorithms did not have a big number of examples of alternatives to learn from). Sensitivity is the same in the earlier depressed group, but drops to 52% in the never depressed group, underlining the difficulty in identifying women at high risk for having their first ever depressive episode after childbirth. In general, the high NPV figure in the never earlier depressed group means that women with a negative screening in that group do not need tighter follow-up; NPV nonetheless drops to 86% in the earlier depressed group, suggesting that further screening in the postpartum period might still benefit this high-risk group of women.

Our study showed a slightly higher AUC than most earlier studies’ best prediction models (79% by Wang et al. and 78% by Zhang et al.), though our accuracy of 73% is lower than the 84% reported by Tortajada et al.^34,36,38^. However, in the latter study, the main outcome was depression at 32 weeks and not at 6 weeks postpartum, genetic data was included and the study sample was more homogeneous since it consisted of SSRI-free Caucasian women. Moreover, a lower EPDS cut-off was used followed by clinical interviews, possibly reducing the risk of misclassification of study cases and controls. Nevertheless, in our study, a clinical evaluation was not possible for practical reasons, due to the much larger study population. Finally, in addition to clinical and environmental variables, information on related gene polymorphisms was also utilized in that study.

Furthermore, Wang et al. identified race, obesity, anxiety, depression, different types of pain, and antidepressant and anti-inflammatory drug use during pregnancy as the most important variables for their prediction models^34^. These variables differed somewhat from the ones we identified as being most important with the caveat that our model also indicated that anxiety during pregnancy and depression history or depressive symptoms during pregnancy were overwhelmingly the most significant predictors for PPD. It has to be noted that we included many psychometric measures, which followed in importance, e.g. the question 19 on the SOC scale “Do you have very mixed up feelings and ideas?” and question 4 on RS, which measures self-regard (“I am friends with myself”). The population in the BASIC study is quite homogeneous, most participants having a high education, are quite healthy and born in the Nordic countries. Further, the BASIC dataset has no information on race. BMI was also identified in our study as an important variable, both in the BP dataset analysis and the sub-analysis among women without previous depression. Rates of antidepressant use are low. Differences in the analytical approach might also account for some differences in the results.

These findings further illuminate the difficulties in predicting which women will go on to develop postpartum depressive symptoms after childbirth. From the variable importance plots, the most predictive variables for postpartum depressive symptoms, available at the time of discharge from the delivery ward, is to either have anxiety or depressive symptoms during pregnancy. In fact, these two variables are by far the most predictive, along nevertheless with distinct variables related to resilience, sense of coherence and personality. The predictive algorithms reach an accuracy for the whole group of 73% and AUC of 81%, which is at the limit for possible use in clinical settings. The algorithms might need to be different according to whether women had experience depression before in life. Further studies, possibly using more advanced methods and bigger samples, are warranted.

Very recently, Zhang et al. also proposed a machine learning based framework for PPD risk prediction using electronic health record (EHR) data^39^. While the techniques employed are comparable to our study with similar processing pipeline, they report higher AUC. This increment can be attributed majorly to the substantially larger cohort used in their study. Several ML studies have demonstrated that large datasets lead to lower estimation variance and hence provide better predictive performance. Furthermore, the top predictors also differ between our study due to differences in data sources. Additionally, a PPV higher than that reported in our study would significantly increase the clinical utility of our proposed framework. However, PPV is directly related to the prevalence of PPD in the population studied, which is only about 12%. While the classification threshold of the model can be adjusted to improve PPV, it does not ensure the expected benefit as other evaluation metrics, like sensitivity, specificity and NPV, would be adversely affected. Even Zhang et al. that reported higher AUC values, only report a PPV of ~ 27% for the validation site with prevalence of 6.5%, highlighting the issue^39^.

The lack of effective ways that would allow for early prediction of women at risk for depressive symptoms in the postpartum period has been addressed in the Introduction. In fact, the Edinburgh Postnatal Depression Scale is nowadays used as a screening tool for current depression^41^. National guidelines in several countries recommend screening for PPD at 6 to 8 weeks postpartum; however, the suggested target groups of women to be screened vary between countries^42–44^. Also, the use of the EPDS at this time is used to screen for concurrent depression. In contrast, the role of EPDS in pregnancy, in combination with other variables, for early identification of women at risk for development of depressive symptoms later in the postpartum period has not been studied. In our study we do show that high EPDS scores in pregnancy are highly predictive of postpartum depression.

This study had numerous strengths. First, it addresses a novel field, as there are very few studies in the area, none from the Nordic countries, and none of earlier algorithms is being widely used in clinical practice. The large sample size allowed us to train a robust range of different ML algorithms. The richness of the BASIC dataset provided us with the opportunity to investigate the predictive power of a large number of background, medical history, pregnancy and delivery related variables, as well as psychometric questionnaires; the last ones both as total scores but also at individual item level. A key novelty feature of the study in the inclusion of many resilience and personality-related variables, that have been identified in the literature but not included in previous models. We also explore the importance of variables in terms of their predictive power of PPD, an effort directed towards to designing a compact survey to screen for PPD. Finally, the analysis of clinically relevant sub-groups such as women with previous depression or depression during pregnancy gave clinically useful insights.

Some limitations of the study include the non-representative sample in that women born in Scandinavia, with a high education and cohabitating with the child’s father were over-represented in the cohort, which makes the findings difficult to generalize to the background population. Sources of selection bias are the exclusion of non-Swedish speaking women as the questionnaires were only offered in the Swedish language, and the fact that more healthy women are more prone to participate in studies of this kind. Not all women self-reported on all variables, but we addressed this problem of missing values with exclusions and imputations where appropriate. Class imbalance in the outcome made the training stages of the algorithms challenging but were also addressed appropriately. Lastly, theoretically, some items from the scales on personality (SSP), and attachment (ASQ) might have had a more prominent role in prediction if they would have been available for a larger proportion of the women in this study. The study by Zhang et al., published after our study was conducted, reported higher AUC and included some predictors lacking in our study^39^. Future studies should make sure to include these important predictive variables for further evaluation.

Depressive symptoms and anxiety during pregnancy are highly predictive factors for women who go on and develop postpartum depressive symptoms, while variables relating to resilience, sense of coherence and personality also play a modest role. The predictive algorithms have relatively good accuracy and AUC, with XRT performing best.

## Methods

### Data sources

Data for the development of the prediction models were obtained from the “Biology, Affect, Stress, Imaging and Cognition during Pregnancy and the Puerperium” (BASIC) study. BASIC is a population-based prospective cohort study at the Department of Obstetrics and Gynaecology at Uppsala University Hospital, Uppsala, Sweden^7^. Between September 2009 and November 2018 all pregnant women who were 18 years of age or older, did not have their identities concealed, had sufficient ability to read and understand Swedish and did not have known bloodborne infections and/or non-viable pregnancy as diagnosed by routine ultrasound were invited to participate in the study^45^. Data acquisition in the BASIC study was mainly based on online surveys and questionnaires that the women were asked to fill out during pregnancy at the 17th and 32nd gestational week and at 6 weeks, 6 months and 12 months postpartum. The surveys included questions about background characteristics, such as sociodemographic variables, psychological measures, medical information, information on reproductive history, lifestyle and sleep. All questionnaires were self-reported and web-based. Data are also retrieved from the medical journals. The participation rate for the study was 20% but the cohort had a relatively low attrition rate, with 71% of the participants remaining in the study at 12 months follow-up^45^.

This study focuses on two subsets of variables from the BASIC study: and (i) background, medical history and pregnancy/delivery variables (BP) and (ii) further psychometric questionnaires (information on exact assessment methods and coding is provided in Table 1 for the background variables and Supplementary Table 1 for the exact questions in the different questionnaires). The BP variables consisted of sociodemographic and lifestyle information, self-reported health, medical history and variables relating to pregnancy and childbirth. This dataset included even information on depression and anxiety symptoms during pregnancy. Depression symptoms were assessed by a score of 12 or more on the Edinburg Postnatal Depression Scale (EPDS) in pregnancy weeks 17, 32 or 38, while anxiety during pregnancy was defined as ratings in the highest quartile on either the State Trait Anxiety Inventory (STAI)^46^, the Beck Anxiety Inventory or the anxiety subscale of the EPDS (EPDS-3A). These variables were available for the majority of the BASIC participants. The total number of interpersonal and non-interpersonal events in the Lifetime Instances of Traumatic Events Scale (LITE)^47^ was also included among BP variables. The BP variables consisted of continuous, discrete, nominal and ordinal categorical variables, measured at various time points during the study.

The extra psychometric scales used were the Attachment Style Questionnaire (ASQ)^48^, the Resilience-14 scale (RS)^49,50^, the Sense of Coherence Scale-29 (SOC)^51^, the Vulnerable Personality Style Questionnaire (VPSQ)^52,53^, and the Swedish Scale of Personalities (SSP)^54^. ASQ, RS, SOC, VPSQ, and SSP were filled out at gestational week 17 or 32, VPSQ and LITE assessments were conducted at 12 months postpartum. All variables were assessed on a Likert scale and coded as ordinal variables. These scales were used for only specific period of time during the course of the BASIC project, different for each scale, and are thus available for different number of women (Table 1)^45^.

Additionally, the participants of BASIC study were also asked to fill out the EPDS at different time-points during and after pregnancy. The outcome in this study was EPDS score at 6 weeks postpartum, assessing the degree of self-reported depressive symptoms in the early postpartum period. The discrete scores for this timepoint were then aggregated and a cut-off of a score of 12 or higher was used to indicate women with depressive symptoms, in accordance to validation studies for the Swedish population^55^. The number of women in the BASIC study who had completed the EPDS at 6 weeks postpartum and were thus included was 4313.

### Ethics declarations

The study has been approved by the Research Ethics Board in Uppsala (Dnr 2009/171, with amendments). All participating women gave written informed consent before being included in the study. All methods were carried out in accordance with relevant guidelines and regulations.

### Data pre-processing

The pre-processing consisted of splitting the original BASIC dataset into different subsets. Two subsets were retained for our study, i.e. background & pregnancy (BP) data and psychometric questionnaire data. Data for twins and women with multiple pregnancies were removed from the dataset, as these are relatively rare, are followed very closely during and after childbirth, and are associated with higher risk for PPD^56,57^. Explorative data analyses were conducted on individual variables to check their distributions and to identify and remove outliers that were assessed to be non-informative. Psychometric questionnaires and BP variables that contained information about the women after the time point of the outcome, namely 6 weeks postpartum, were also excluded to avoid inadvertent biases of the results.

SSP was omitted from the analysis due to large number of missing observations, as this survey was used only for few years during recruitment for the BASIC study^45^. Its inclusion would have resulted in a much smaller sample size for the final analysis.

The dataset consists of continuous, nominal and ordinal variables. As continuous variables in the dataset have varying scales, normalization is performed to transform all the variables to a common range from 0 to 1. Furthermore, nominal and ordinal variables that represent non-numerical values are encoded using binary numerical representations for improving the performance of the ML algorithms.

### Data imputation

As missing values can drastically impact the performance of ML models, a conservative approach was adopted to handle them. Firstly, samples (rows, corresponding to one pregnancy) with more than 50% missing values in the included variables were eliminated, and the final number of pregnancies in the ML analyses was 4277. Next, variables (columns, corresponding to a distinct variable) with more than 25% missing data were also eliminated. Finally, the remaining missing values were imputed from the available data. While continuous variables were imputed using multivariate imputation by chained equations (MICE)^58^, categorical and ordinal variables were imputed with K nearest neighbors’ imputation^59^.

## Modeling

### Classification techniques

With ML algorithms, there is no one-size-fits-all solution, making it imperative to try multiple alternatives. Consequently, this study explored different ML algorithms for supervised classification that modeled data in different ways. In order to present a comprehensive comparison, the following algorithms were implemented: Ridge Regression, LASSO Regression, Gradient Boosting Machines, Distributed Radom Forests, Extreme Randomized Forest, Naïve Bayes, and Stacked Ensembles. Ridge Regression specializes in analysing multiple regression data with multicollinearity, while LASSO Regression is a type of linear regression that shrinks data values towards a central point, and results in simple, sparse models (i.e. models with fewer parameters). Gradient Boosting Machines (GBM) and Random Forests are ensemble learners. In Distributed Radom Forests (DRF), a subset of features is used to determine the most discriminative thresholds to split the trees on. However, unlike DRF, where one builds an ensemble of deep independent trees, in GBM, we specify an ensemble of weak, shallow successive trees, where each tree is learning and improving on the previous tree. In Extremely Randomized Trees (XRT), instead of using the most discriminative thresholds for the splits, thresholds are drawn at random for each feature and the best of these random thresholds are used as the splitting rule, resulting in lower variance but more bias. XRT are similar to DRF with the caveat of more randomness. Naïve Bayes (NB) is a probabilistic classifier based on Bayes’ Theorem. The NB works under the assumption that the presence of any particular feature for a certain outcome is unrelated to the presence of any other feature for that outcome. Thus, despite if the features depend on each other or upon the existence of other features, the NB assumes that all of the features independently contribute to the outcome probability. Stacked Ensemble learns a new model by combining predictions of existing models. Stacked Ensembles are a class of supervised learning algorithms that work by training a meta-learner to find the optimal combination of base learners. Unlike bagging and boosting were the goal is to stack a number of weak learners together, the goal is to stack a number of diverse and strong learners together to optimize learning^60^.

For all the classification algorithms, the outcome measure was the participants’ EPDS score at 6 weeks postpartum represented as a binary variable with 12 as cut-off, while predictor variables included the BP variables and psychometric data described above.

### Class imbalance

The BASIC dataset, as a population-based sample and in accordance to clinical situations, is predominantly composed of data from women who did not experience PPD at 6 weeks postpartum (less than 10% of the women representing PPD cases), consequently leading to extreme data class imbalance. ML classifiers trained on such imbalanced datasets usually generate biased results. To mitigate this imbalance, the minority class consisting of women with PPD was oversampled during ML training. Unlike under sampling of majority class consisting of women without PPD, this approach avoids loss of information and leverages all the samples from both classes.

### Evaluation metrics

The performance of model prediction of the ML classification algorithms was evaluated using a variety of performance metrics. The performance of each classification model was captured by the Confusion Matrix that formed the basis for other metrics. In addition to the most commonly used classification accuracy, sensitivity (true positive rate) and specificity (false positive rate) are also reported. The positive predictive value (PPV) and negative predictive value (NPV) are also reported. Additionally, a Receiver Operating Characteristic (ROC) curve was specified for each classification to show the relation between the true positive rate and false positive rate. The performance of the classifiers was then summarized by the total area under the ROC curve (AUC), with the higher the AUC (between 0 and 1) indicating a better performance of the classification.

### Variable (feature) importance/selection

The success of a ML algorithm does not only depend on good predictive performance but also on generalizability and easy interpretability. Identifying variables that have significant impact on the outcome is valuable, especially in the medical domain. Variable importance using Random Forests models can be calculated using Gini Importance or Mean Decrease in Impurity (MDI)^61^. The MDI relevance of a variable is obtained by calculating how effective the variable is at reducing the uncertainty when creating decision trees. The variable that is most effective and used the most will be ranked as most important.

### Analytic strategy

**Figure 7:**
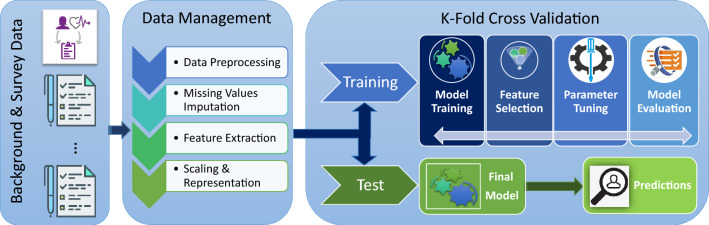
Study workflow and analytical strategy. Data were obtained from the “Biology, Affect, Stress, Imaging and Cognition during Pregnancy and the Puerperium” (BASIC) study, a population-based prospective cohort study in Uppsala, Sweden. Data included in our study comprised (i) background, medical history and pregnancy-related variables (BP) from women, and (ii) further psychometric questionnaires, available at discharge from the delivery ward. The data were processed and either were used to test models or train the machine learning algorithms, to predict depressive symptoms at 6 weeks postpartum.

The analytical strategy consisted of breaking the analysis down into steps and iteratively building towards a final classification model, all the while being cognizant of any potential biases introduced by the approach. The workflow is presented in Fig. 7. First, the raw data was split into the BP and the different psychometric questionnaires datasets in order to build predictive models independently on each psychometric questionnaire and to identify the ones with the highest accuracy for classification of PPD. Second, the psychometric questionnaires that yielded the highest accuracies were combined with the BP dataset. Predictions were then performed with the aggregate data (combined dataset). Additional models were trained with reduced datasets resulting from variable selection. Top 50% and top 25% variables with MDI were used to train separate classification models to determine the relative contribution of those variables to the prediction. Additionally, stratified analyses were performed, where participants were stratified by a previous history of depression (defined as earlier depression, earlier contact with psychiatrist/psychologist, or depression during pregnancy).

Based on preliminary analyses, SSP and ASQ did not provide any information gain relative to BP data. Hence, only RS, SOC and VPSQ variables that provided predictive performances comparable to BP variables were included in the aggregate analysis.

## Supplementary Information

Supplementary Informations.

## Data Availability

The datasets generated and/or analysed during the current study are available from the corresponding author on reasonable request and after data transfer agreements are in place, according to current regulations.

## References

[1] American Psychiatric Association (2013). Diagnostic and Statistical Manual of Mental Disorders: DSM-5.

[2] Fitelson E, Kim S, Baker AS, Leight K (2010). Treatment of postpartum depression: clinical, psychological and pharmacological options. Int. J. Womens Health.

[3] Patel M (2012). Postpartum depression: a review. J. Health Care Poor Underserved.

[4] Yim IS, Tanner Stapleton LR, Guardino CM, Hahn-Holbrook J, Dunkel Schetter C (2015). Biological and psychosocial predictors of postpartum depression: systematic review and call for integration. Annu. Rev. Clin. Psychol..

[5] Bloch M, Daly RC, Rubinow DR (2003). Endocrine factors in the etiology of postpartum depression. Compr. Psychiatry.

[6] Asif S (2020). Severe obstetric lacerations associated with postpartum depression among women with low resilience—a Swedish birth cohort study. BJOG.

[7] Iliadis SI (2015). Personality and risk for postpartum depressive symptoms. Arch. Womens Ment. Health.

[8] Committee on Obstetric Practice (2015). American College of Obstetricians and Gynecologists Committee opinion no. 630: screening for perinatal depression. Obstet. Gynecol..

[9] Ko JY, Rockhill KM, Tong VT, Morrow B, Farr SL (2017). Trends in postpartum depressive symptoms—27 States, 2004, 2008, and 2012. MMWR Morb. Mortal Wkly. Rep..

[10] Dennis CL, McQueen K (2009). The relationship between infant-feeding outcomes and postpartum depression: a qualitative systematic review. Pediatrics.

[11] Slomian J, Honvo G, Emonts P, Reginster J-Y, Bruyère O (2019). Consequences of maternal postpartum depression: a systematic review of maternal and infant outcomes. Womens Health.

[12] Moore Simas TA (2020). Matched cohort study of healthcare resource utilization and costs in young children of mothers with postpartum depression in the United States. J. Med. Econ..

[13] Murray L, Woolgar M, Cooper P, Hipwell A (2001). Cognitive vulnerability to depression in 5-year-old children of depressed mothers. J. Child Psychol. Psychiatry.

[14] Nguyen J (2017). A literature review of alternative therapies for postpartum depression. Nurs. Womens Health.

[15] The Management of Depression During Pregnancy (2009). A report from the American Psychiatric Association and the American College of Obstetricians and Gynecologists. Obstet. Gynecol..

[16] Dennis C-L, Hodnett ED (2007). Psychosocial and psychological interventions for treating postpartum depression. Cochrane Database Syst. Rev..

[17] Righetti-Veltema M, Conne-Perréard E, Bousquet A, Manzano J (1998). Risk factors and predictive signs of postpartum depression. J. Affect. Disord..

[18] Lee Y (2018). Applications of machine learning algorithms to predict therapeutic outcomes in depression: a meta-analysis and systematic review. J. Affect. Disord..

[19] Bzdok D, Meyer-Lindenberg A (2018). Machine learning for precision psychiatry: opportunities and challenges. Biol. Psychiatry Cognit. Neurosci. Neuroimaging.

[20] Friston KJ, Redish AD, Gordon JA (2017). Computational nosology and precision psychiatry. Comput. Psychiatry.

[21] Rohart F, Gautier B, Singh A, Lê Cao K-A (2017). mixOmics: an R package for ‘omics feature selection and multiple data integration. PLOS Comput. Biol..

[22] Ahmed FE (2005). Artificial neural networks for diagnosis and survival prediction in colon cancer. Mol. Cancer.

[23] Anagnostou T, Remzi M, Lykourinas M, Djavan B (2003). Artificial neural networks for decision-making in urologic oncology. Eur. Urol..

[24] Jerez J (2005). Improvement of breast cancer relapse prediction in high risk intervals using artificial neural networks. Breast Cancer Res. Treat..

[25] Suzuki K, Li F, Sone S, Doi K (2005). Computer-aided diagnostic scheme for distinction between benign and malignant nodules in thoracic low-dose CT by use of massive training artificial neural network. IEEE Trans. Med. Imaging.

[26] Baxt WG, Shofer FS, Sites FD, Hollander JE (2002). A neural computational aid to the diagnosis of acute myocardial infarction. Ann. Emerg. Med..

[27] Zini G (2005). Artificial intelligence in hematology. Hematology.

[28] Bent P (2001). Early and intensive continuous hemofiltration for severe renal failure after cardiac surgery. Ann. Thorac. Surg..

[29] Huang L, Yu P, Ju F, Cheng J (2003). Prediction of response to incision using the mutual information of electroencephalograms during anaesthesia. Med. Eng. Phys..

[30] Choi J, Choi J, Jung H-T (2018). Applying machine-learning techniques to build self-reported depression prediction models. CIN Comput. Inform. Nurs..

[31] Gao S, Calhoun VD, Sui J (2018). Machine learning in major depression: from classification to treatment outcome prediction. CNS Neurosci. Ther..

[32] Graham S (2019). Artificial intelligence for mental health and mental illnesses: an overview. Curr. Psychiatry Rep..

[33] Helbich M, Hagenauer J, Roberts H (2019). Relative importance of perceived physical and social neighborhood characteristics for depression: a machine learning approach. Soc. Psychiatry Psychiatr. Epidemiol..

[34] Wang S, Pathak J, Zhang Y (2019). Using electronic health records and machine learning to predict postpartum depression. Stud. Health Technol. Inform..

[35] Tai AMY (2019). Machine learning and big data: implications for disease modeling and therapeutic discovery in psychiatry. Artif. Intell. Med..

[36] Tortajada S (2009). Prediction of postpartum depression using multilayer perceptrons and pruning. Methods Inf. Med..

[37] Jiménez-Serrano S, Tortajada S, García-Gómez JM (2015). A mobile health application to predict postpartum depression based on machine learning. Telemed. e-Health.

[38] Zhang W, Liu H, Silenzio VMB, Qiu P, Gong W (2020). Machine learning models for the prediction of postpartum depression: application and comparison based on a cohort study. JMIR Med. Inform..

[39] Zhang Y, Wang S, Hermann A, Joly R, Pathak J (2021). Development and validation of a machine learning algorithm for predicting the risk of postpartum depression among pregnant women. J. Affect. Disord..

[40] Luca DL, Garlow N, Staatz C, Margiotta C, Zivin K (2019). Societal Costs of Untreated Perinatal Mood and Anxiety Disorders in the United States.

[41] Levis B, Negeri Z, Sun Y, Benedetti A, Thombs BD (2020). Accuracy of the Edinburgh Postnatal Depression Scale (EPDS) for screening to detect major depression among pregnant and postpartum women: systematic review and meta-analysis of individual participant data. BMJ.

[42] Siu AL, Force, A. T. U. P. S. T (2016). Screening for depression in adults: US Preventive Services Task Force recommendation statement. JAMA.

[43] National Collaborating Centre for Mental Health (UK). *Antenatal and Postnatal Mental Health: Clinical Management and Service Guidance: Updated Edition* (NICE Clinical Guidelines, No. 192. 5, CASE IDENTIFICATION AND ASSESSMENT, British Psychological Society, 2014). 26180865

[44] Austin MP, Highet N, Group EW (2017). Mental Health Care in the Perinatal Period: Australian Clinical Practice Guideline.

[45] Axfors C (2019). Cohort profile: the Biology, Affect, Stress, Imaging and Cognition (BASIC) study on perinatal depression in a population-based Swedish cohort. BMJ Open.

[46] Spielberger, C. D. State‐trait anxiety inventory. In *The Corsini Encyclopedia of Psychology*, 1. 10.1002/9780470479216.corpsy0943 (2010).

[47] Greenwald R, Rubin A (1999). Assessment of posttraumatic symptoms in children: development and preliminary validation of parent and child scales. Res. Soc. Work Pract..

[48] Feeney, J., Noller, P. & Hanrahan, M. Assessing adult attachment. In *Attachment in Adults: Clinical and Developmental Perspectives* (eds Sperling, M. B. & Berman, W. H.) 128–151 (The Guilford Press, New York, 1994).

[49] Aiena BJ, Baczwaski BJ, Schulenberg SE, Buchanan EM (2015). Measuring resilience with the RS–14: a tale of two samples. J. Pers. Assess..

[50] Wagnild GM, Young HM (1993). Development and psychometric evaluation of the resilience scale. J. Nurs. Meas..

[51] Antonovsky A (1993). The structure and properties of the sense of coherence scale. Soc. Sci. Med..

[52] Boyce P, Hickey A, Gilchrist J, Talley NJ (2001). The development of a brief personality scale to measure vulnerability to postnatal depression. Arch. Womens Ment. Health.

[53] Gelabert E (2011). The vulnerable personality style questionnaire: psychometric properties in Spanish postpartum women. Arch. Womens Ment. Health.

[54] Gustavsson JP (2000). Swedish universities Scales of Personality (SSP): construction, internal consistency and normative data. Acta Psychiatr. Scand..

[55] Wickberg B, Hwang CP (1996). The Edinburgh Postnatal Depression Scale: validation on a Swedish community sample. Acta Psychiatr. Scand..

[56] Vilska S (2009). Mental health of mothers and fathers of twins conceived via assisted reproduction treatment: a 1-year prospective study. Hum. Reprod..

[57] Wenze SJ, Battle CL, Tezanos KM (2015). Raising multiples: mental health of mothers and fathers in early parenthood. Arch. Womens Ment. Health.

[58] Azur MJ, Stuart EA, Frangakis C, Leaf PJ (2011). Multiple imputation by chained equations: what is it and how does it work?: Multiple imputation by chained equations. Int. J. Methods Psychiatr. Res..

[59] Beretta L, Santaniello A (2016). Nearest neighbor imputation algorithms: a critical evaluation. BMC Med. Inform. Decis. Mak..

[60] van der Laan MJ, Polley EC, Hubbard AE (2007). Super learner. Stat. Appl. Genet. Mol. Biol..

[61] Hastie T, Tibshirani R, Friedman JH (2009). The Elements of Statistical Learning: Data Mining, Inference, and Prediction.

