# Supplementary material for: Predicting women with depressive symptoms postpartum with machine learning methods
Source: Sci Rep. 2021 Apr 12;11:7877. doi: 10.1038/s41598-021-86368-y (PMC8041863; doi:10.1038/s41598-021-86368-y)
Supplement: Supplementary file 1 — Supplementary Informations. [file 41598_2021_86368_MOESM1_ESM.pdf]

Predicting Women with Depressive Symptoms Postpartum with Machine Learning Methods

Sam Andersson[1], Deepti R. Bathula[2], Stavros I. Iliadis[1], Martin Walter[3-5], Alkistis Skalkidou[1*]

[1] Department of Women's and Children's Health, Uppsala University, 751 85, Uppsala, Sweden
[2] Department of Computer Science & Engineering, Indian Institute of Technology Ropar, Punjab - 140001, India
[3] Department of Psychiatry and Psychotherapy, University Hospital Jena, Jena, Germany
[4] Department of Psychiatry and Psychotherapy, Eberhardt Karls University, Tübingen, Germany
[5] Department of Behavioral Neurology, Leibniz Institute for Neurobiology, Magdeburg, Germany

Supplementary Table 1. Questionnaire items

**SOC 1 (Sense of Coherence scale-29) [1]**	When you talk to people, do you have the feeling that they don´t understand you?	Ordinal	1 Never have this feeling<br>2<br>3<br>4<br>5<br>6<br>7 Always have this feeling
**SOC 2**	In the past, when you had to do something which depended upon cooperation with others, did you have the feeling that it:	Ordinal	1 Surely woudn't get done<br>2<br>3<br>4<br>5<br>6<br>7 Sure would get done
**SOC 3**	Think of the people with whom you come into contact daily, aside from the ones to whom you feel closest. How well do you know most of them?	Ordinal	1 You feel that they're strangers<br>2<br>3<br>4<br>5<br>6<br>7 You know them very well

**SOC 4**	Do have the feeling that you don't really care about what goes on around you	Ordinal	1 Very seldom or never<br>2<br>3<br>4<br>5<br>6<br>7 Very often
**SOC 5**	Has it happened in the past that you were surprised by the behavior of people whom you thought you knew well?	Ordinal	1 Never happened<br>2<br>3<br>4<br>5<br>6<br>7 Always happened
**SOC 6**	Has it happened that people whom you counted on disappointed you?	Ordinal	1 Never happened<br>2<br>3<br>4<br>5<br>6<br>7 Always happened
**SOC 7**	Life is:	Ordinal	1 Full of interest<br>2<br>3<br>4<br>5<br>6<br>7 Completely routine
**SOC 8**	Until now your life has had:	Ordinal	1 No clear goals or purpose at all<br>2<br>3<br>4<br>5<br>6<br>7 Very clear goals and purpose

**SOC 9**	Do you have the feeling that you're being treated unfairly?	Ordinal	1 Very often<br>2<br>3<br>4<br>5<br>6<br>7 Very seldom or never
**SOC 10**	In the past ten years your life has been:	Ordinal	1 Full of changes without your knowing what will happen next<br>2<br>3<br>4<br>5<br>6<br>7 Completely consistent and clear
**SOC 11**	Most of the things you do in the future will probably be:	Ordinal	1 Completely fascinating<br>2<br>3<br>4<br>5<br>6<br>7 Deadly boring
**SOC 12**	Do you have the feeling that you are in an unfamiliar situation and don't know what to do?	Ordinal	1 Very often<br>2<br>3<br>4<br>5<br>6<br>7 Very seldom or never
**SOC 13**	What best describes how you see life:	Ordinal	1 One can always find a solution to painful things in life<br>2<br>3<br>4<br>5<br>6<br>7 There is no

			solution to painful things in life
**SOC 14**	When you think about your life, you very often:l	Ordinal	1 Feel how good it is to be alive<br>2<br>3<br>4<br>5<br>6<br>7 Ask yourself why you exist at all
**SOC 15**	When you face a difficult problem, the choice of a solution is:	Ordinal	1 Always confusing and hard to find<br>2<br>3<br>4<br>5<br>6<br>7 Always completely clear
**SOC 16**	Doing the things you do every day is:	Ordinal	1 A source of deep pleasure and satisfaction<br>2<br>3<br>4<br>5<br>6<br>7 A source of pain and boredom
**SOC 17**	Your life in the future will probably be:	Ordinal	1 Full of changes without your knowing what will happen next<br>2<br>3<br>4<br>5<br>6

			7 Completely consistent and clear
**SOC 18**	When something unpleasant happened in the past your tendency was:	Ordinal	1 “to eat yourself up about it”<br>2<br>3<br>4<br>5<br>6<br>7 to say “ok that’s that, I have to live with it” and go on
**SOC 19**	Do you have very mixed-up feelings and ideas?	Ordinal	1 Very often<br>2<br>3<br>4<br>5<br>6<br>7 Very seldom or never
**SOC 20**	When you do something that gives you a good feeling:	Ordinal	1 It’s certain that you’ll go on feeling good<br>2<br>3<br>4<br>5<br>6<br>7 It’s certain that something will happen to spoil the feeling
**SOC 21**	Does it happen that you have feelings inside you would rather not feel?	Ordinal	1 Very often<br>2<br>3<br>4<br>5<br>6<br>7 Very seldom or never

**SOC 22**	You anticipate that your personal life in the future will be:	Ordinal	1 Totally without meaning or purpose<br>2<br>3<br>4<br>5<br>6<br>7 Full of meaning and purpose
**SOC 23**	Do you think that there will always be people whom you'll be able to count on in the future?	Ordinal	1 You're certain there will be<br>2<br>3<br>4<br>5<br>6<br>7 You doubt there will be
**SOC 24**	Does it happen that you have the feeling that you don't know exactly what's about to happen?	Ordinal	1 Very often<br>2<br>3<br>4<br>5<br>6<br>7 Very seldom or never
**SOC 25**	Many people - even those with a strong character - sometimes feel like sad sacks (losers) in certain situations. How often have you felt this way in the past?	Ordinal	1 Never<br>2<br>3<br>4<br>5<br>6<br>7 Very often
**SOC 26**	When something happened, have you generally found that:	Ordinal	1 You overestimated or underestimated its importance<br>2<br>3<br>4<br>5<br>6<br>7 You saw things in the right proportion

**SOC 27**	When you think of the difficulties you are likely to face in important aspects of your life, do you have the feeling that:	Ordinal	1 You will always succeed in overcoming the difficulties<br>2<br>3<br>4<br>5<br>6<br>7 You won't succeed in overcoming the difficulties
**SOC 28**	How often do you have the feeling that there's little meaning in the things you do in your daily life?	Ordinal	1Very often<br>2<br>3<br>4<br>5<br>6<br>7 Very seldom or never
**SOC 29**	How often do you have feelings that you're not sure you can keep under control?	Ordinal	1 Very often<br>2<br>3<br>4<br>5<br>6<br>7 Very seldom or never
**Resilience scale-14**<br>**RS 1: Ability to Cope** [2,3]<br>(43,54)	I usually manage one way or another.	Ordinal	1 Completely disagree<br>2<br>3<br>4<br>5<br>6<br>7 Completely agree
**RS 2: Pride**	feel proud that I have accomplished things in life.	Ordinal	1 Completely disagree<br>2<br>3<br>4<br>5<br>6<br>7 Completely agree

**RS 3: Acceptance**	I usually take things in stride.	Ordinal	1 Completely disagree<br>2<br>3<br>4<br>5<br>6<br>7 Completely agree
**RS 4: Self-regard**	I am friends with myself.	Ordinal	1 Completely disagree<br>2<br>3<br>4<br>5<br>6<br>7 Completely agree
**RS 5: Organized**	I feel that I can handle many things at a time.	Ordinal	1 Completely disagree<br>2<br>3<br>4<br>5<br>6<br>7 Completely agree
**RS 6: Drive**	I am determined.	Ordinal	1 Completely disagree<br>2<br>3<br>4<br>5<br>6<br>7 Completely agree
**RS 7: Perseverance**	I can get through difficult times because I've experienced difficulty before.	Ordinal	1 Completely disagree<br>2<br>3<br>4<br>5<br>6<br>7 Completely agree

**RS 8: Willpower**	I have self-discipline.	Ordinal	1 Completely disagree<br>2<br>3<br>4<br>5<br>6<br>7 Completely agree
**RS 9: Interest/engagement**	I keep interested in things.	Ordinal	1 Completely disagree<br>2<br>3<br>4<br>5<br>6<br>7 Completely agree
**RS 10: Humor**	I can usually find something to laugh about.	Ordinal	1 Completely disagree<br>2<br>3<br>4<br>5<br>6<br>7 Completely agree
**RS 11: Self-efficacy**	My belief in myself gets me through hard times.	Ordinal	1 Completely disagree<br>2<br>3<br>4<br>5<br>6<br>7 Completely agree
**RS 12: Dependable**	In an emergency, I'm someone people can generally rely on.	Ordinal	1 Completely disagree<br>2<br>3<br>4<br>5<br>6<br>7 Completely agree

**RS 13: Meaning**	My life has meaning.	Ordinal	1 Completely disagree<br>2<br>3<br>4<br>5<br>6<br>7 Completely agree
**RS 14: Resourcefulness**	When I am in a difficult situation, I can usually find my way out of it.	Ordinal	1 Completely disagree<br>2<br>3<br>4<br>5<br>6<br>7 Completely agree
**RS 16:**	I can usually find something to laugh about.	Ordinal	1 Completely disagree<br>2<br>3<br>4<br>5<br>6<br>7 Completely agree
**Attachment Scale Questionnaire ASQ 1** [4]	worth getting to know.	Ordinal	1 Completely disagree<br>2 Disagree a lot<br>3 Somewhat disagree<br>4 Somewhat agree<br>5 Agree a lot<br>6 Agree completely
**ASQ 2**	easier to get to know than most others.	Ordinal	1 Completely disagree<br>2 Disagree a lot<br>3 Somewhat disagree<br>4 Somewhat agree<br>5 Agree a lot<br>6 Agree completely

**ASQ 3**	sure others will be on hand for me when I need them.	Ordinal	1 Completely disagree<br>2 Disagree a lot<br>3 Somewhat disagree<br>4 Somewhat agree<br>5 Agree a lot<br>6 Agree completely
**ASQ 4**	myself than to other people.	Ordinal	1 Completely disagree<br>2 Disagree a lot<br>3 Somewhat disagree<br>4 Somewhat agree<br>5 Agree a lot<br>6 Agree completely
**ASQ 5**	keep me to myself.	Ordinal	1 Completely disagree<br>2 Disagree a lot<br>3 Somewhat disagree<br>4 Somewhat agree<br>5 Agree a lot<br>6 Agree completely
**ASQ 6**	ask for help is to admit that you are unsuccessful.	Ordinal	1 Completely disagree<br>2 Disagree a lot<br>3 Somewhat disagree<br>4 Somewhat agree<br>5 Agree a lot<br>6 Agree completely
**ASQ 7**	people's value should be judged on what they achieve.	Ordinal	1 Completely disagree<br>2 Disagree a lot<br>3 Somewhat disagree<br>4 Somewhat agree<br>5 Agree a lot

			6 Agree completely
**ASQ 8**	Achieving things is more important than building relationships.	Ordinal	1 Completely disagree<br>2 Disagree a lot<br>3 Somewhat disagree<br>4 Somewhat agree<br>5 Agree a lot<br>6 Agree completely
**ASQ 9**	Doing their best is more important than getting along with others.	Ordinal	1 Completely disagree<br>2 Disagree a lot<br>3 Somewhat disagree<br>4 Somewhat agree<br>5 Agree a lot<br>6 Agree completely
**ASQ 10**	you should do so even if someone is injured.	Ordinal	1 Completely disagree<br>2 Disagree a lot<br>3 Somewhat disagree<br>4 Somewhat agree<br>5 Agree a lot<br>6 Agree completely
**ASQ 11**	important for me to be liked?	Ordinal	1 Completely disagree<br>2 Disagree a lot<br>3 Somewhat disagree<br>4 Somewhat agree<br>5 Agree a lot<br>6 Agree completely

**ASQ 12**	important for me to avoid doing things that others would not like.	Ordinal	1 Completely disagree<br>2 Disagree a lot<br>3 Somewhat disagree<br>4 Somewhat agree<br>5 Agree a lot<br>6 Agree completely
**ASQ 13**	It's hard to make decisions when I don't know what others think.	Ordinal	1 Completely disagree<br>2 Disagree a lot<br>3 Somewhat disagree<br>4 Somewhat agree<br>5 Agree a lot<br>6 Agree completely
**ASQ 14**	usually superficial.	Ordinal	1 Completely disagree<br>2 Disagree a lot<br>3 Somewhat disagree<br>4 Somewhat agree<br>5 Agree a lot<br>6 Agree completely
**ASQ 15**	I can't do anything.	Ordinal	1 Completely disagree<br>2 Disagree a lot<br>3 Somewhat disagree<br>4 Somewhat agree<br>5 Agree a lot<br>6 Agree completely
**ASQ 16**	It's hard to trust other people.	Ordinal	1 Completely disagree<br>2 Disagree a lot<br>3 Somewhat disagree<br>4 Somewhat agree<br>5 Agree a lot

			6 Agree completely
**ASQ 17**	It's hard to depend on others	Ordinal	1 Completely disagree<br>2 Disagree a lot<br>3 Somewhat disagree<br>4 Somewhat agree<br>5 Agree a lot<br>6 Agree completely
**ASQ 18**	others do not want to come as close to me as I would like them.	Ordinal	1 Completely disagree<br>2 Disagree a lot<br>3 Somewhat disagree<br>4 Somewhat agree<br>5 Agree a lot<br>6 Agree completely
**ASQ 19**	get close to other people.	Ordinal	1 Completely disagree<br>2 Disagree a lot<br>3 Somewhat disagree<br>4 Somewhat agree<br>5 Agree a lot<br>6 Agree completely
**ASQ 20**	It is easy to trust others.	Ordinal	1 Completely disagree<br>2 Disagree a lot<br>3 Somewhat disagree<br>4 Somewhat agree<br>5 Agree a lot<br>6 Agree completely

**ASQ 21**	be dependent on other people.	Ordinal	1 Completely disagree<br>2 Disagree a lot<br>3 Somewhat disagree<br>4 Somewhat agree<br>5 Agree a lot<br>6 Agree completely
**ASQ 22**	others should not care as much about me as I care about them.	Ordinal	1 Completely disagree<br>2 Disagree a lot<br>3 Somewhat disagree<br>4 Somewhat agree<br>5 Agree a lot<br>6 Agree completely
**ASQ 23**	people will come too close to me.	Ordinal	1 Completely disagree<br>2 Disagree a lot<br>3 Somewhat disagree<br>4 Somewhat agree<br>5 Agree a lot<br>6 Agree completely
**ASQ 24**	I should not be as good as other people.	Ordinal	1 Completely disagree<br>2 Disagree a lot<br>3 Somewhat disagree<br>4 Somewhat agree<br>5 Agree a lot<br>6 Agree completely
**ASQ 25**	mixed feelings towards proximity to others.	Ordinal	1,00 Completely disagree<br>2,00 Disagree a lot<br>3,00 Somewhat disagree<br>4,00 Somewhat agree

			5,00 Agree a lot<br>6,00 Agree completely
**ASQ 26**	I feel uneasy about it.	Ordinal	1 Completely disagree<br>2 Disagree a lot<br>3 Somewhat disagree<br>4 Somewhat agree<br>5 Agree a lot<br>6 Agree completely
**ASQ 27**	why people would want to get involved in me.	Ordinal	1 Completely disagree<br>2 Disagree a lot<br>3 Somewhat disagree<br>4 Somewhat agree<br>5 Agree a lot<br>6 Agree completely
**ASQ 28**	have a close relationship.	Ordinal	1 Completely disagree<br>2 Disagree a lot<br>3 Somewhat disagree<br>4 Somewhat agree<br>5 Agree a lot<br>6 Agree completely
**ASQ 29**	my relationships.	Ordinal	1 Completely disagree<br>2 Disagree a lot<br>3 Somewhat disagree<br>4 Somewhat agree<br>5 Agree a lot<br>6 Agree completely

**ASQ 30**	how I would manage without someone who loves me.	Ordinal	1 Completely disagree<br>2 Disagree a lot<br>3 Somewhat disagree<br>4 Somewhat agree<br>5 Agree a lot<br>6 Agree completely
**ASQ 31**	confident in my contact with others	Ordinal	1 Completely disagree<br>2 Disagree a lot<br>3 Somewhat disagree<br>4 Somewhat agree<br>5 Agree a lot<br>6 Agree completely
**ASQ 32**	often outside or alone.	Ordinal	1 Completely disagree<br>2 Disagree a lot<br>3 Somewhat disagree<br>4 Somewhat agree<br>5 Agree a lot<br>6 Agree completely
**ASQ 33**	I don't really fit in with other people.	Ordinal	1 Completely disagree<br>2 Disagree a lot<br>3 Somewhat disagree<br>4 Somewhat agree<br>5 Agree a lot<br>6 Agree completely
**ASQ 34**	I don't bother them with mine.	Ordinal	1 Completely disagree<br>2 Disagree a lot<br>3 Somewhat disagree<br>4 Somewhat agree<br>5 Agree a lot

			6 Agree completely
**ASQ 35**	I mostly feel ashamed or stupid.	Ordinal	1 Completely disagree<br>2 Disagree a lot<br>3 Somewhat disagree<br>4 Somewhat agree<br>5 Agree a lot<br>6 Agree completely
**ASQ 36**	Too busy with other things to spend too much time on relationships.	Ordinal	1 Completely disagree<br>2 Disagree a lot<br>3 Somewhat disagree<br>4 Somewhat agree<br>5 Agree a lot<br>6 Agree completely
**ASQ 37**	Others usually notice it and care about me.	Ordinal	1 Completely disagree<br>2 Disagree a lot<br>3 Somewhat disagree<br>4 Somewhat agree<br>5 Agree a lot<br>6 Agree completely
**ASQ 38**	Other people will like and respect me.	Ordinal	1 Completely disagree<br>2 Disagree a lot<br>3 Somewhat disagree<br>4 Somewhat agree<br>5 Agree a lot<br>6 Agree completely

**ASQ 39**	Others are not available when I need them.	Ordinal	1 Completely disagree<br>2 Disagree a lot<br>3 Somewhat disagree<br>4 Somewhat agree<br>5 Agree a lot<br>6 Agree completely
**ASQ 40**	Other people often disappoint me.	Ordinal	1 Completely disagree<br>2 Disagree a lot<br>3 Somewhat disagree<br>4 Somewhat agree<br>5 Agree a lot<br>6 Agree completely
**Vulnerable Personality Scale Questionnaire VPSQ 1: Coping [5,6]**	1. I would generally describe myself as someone who has difficulty coping with things	Ordinal	1 Not correct at all<br>2<br>3<br>4 Completely correct
**VPSQ 2: Nervy**	2. I would generally describe myself as a nervous / nervous person	Ordinal	1 Not correct at all<br>2<br>3<br>4 Completely correct
**VPSQ 3: Timidity**	3. I would generally describe myself as a shy person who is unable to assert myself	Ordinal	1 Not correct at all<br>2<br>3<br>4 Completely correct
**VPSQ 4: Sensitivity**	4. I am generally the kind of person who worries about what others think of me	Ordinal	1 Not correct at all<br>2<br>3<br>4 Completely correct

**VPSQ 5: Worrier**	5. I usually describe myself as a person who cannot help but worry	Ordinal	1 Not correct at all<br>2<br>3<br>4 Completely correct
**VPSQ 6: Organized**	6. I am generally the kind of person who likes to have order and life	Ordinal	1 Not correct at all<br>2<br>3<br>4 Completely correct
**VPSQ 7: Obsessive**	7. I'm generally the kind of person who often has a hard time finishing things because I spend a lot of time getting things exactly right	Ordinal	1 Not correct at all<br>2<br>3<br>4 Completely correct
**VPSQ 8: Expressive**	8. I am generally a person who is very open with my feelings	Ordinal	1 Not correct at all<br>2<br>3<br>4 Completely correct
**VPSQ 9: Volatility**	9. I am generally the kind of person who often causes emotional outbursts or gets angry and loses control	Ordinal	1 Not correct at all<br>2<br>3<br>4 Completely correct

Supplementary Figure 1.
Area Under the Curve (AUC) corresponding to Figures 3 and 4 in the main text
Performances for seven different machine learning algorithms are presented in the Area Under the Curve (AUC) plots for a) background only and b) combined dataset.

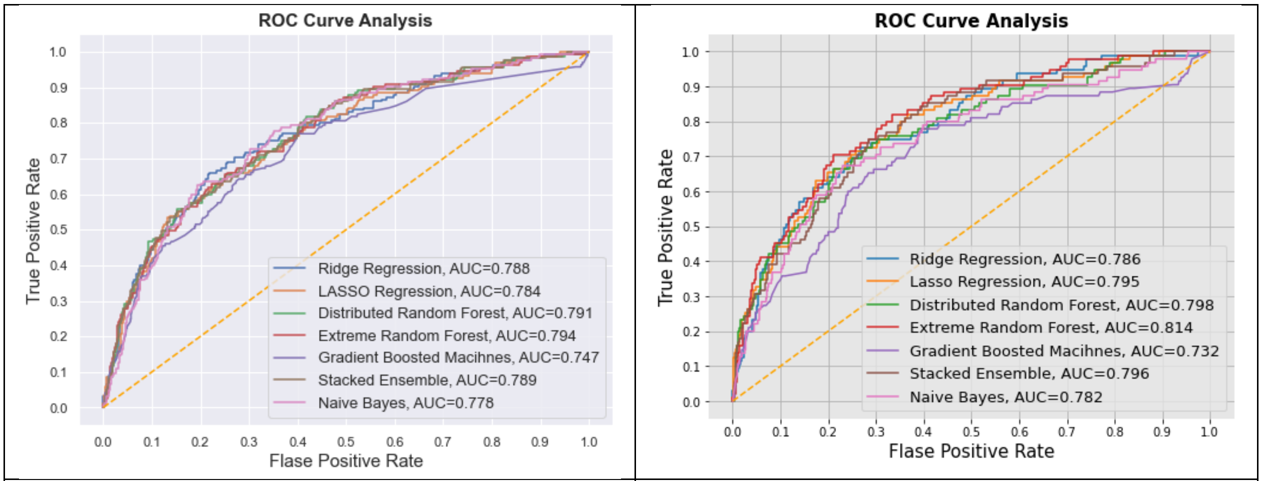

(a) Background only	(b) Combined Dataset

Supplementary Figure 2.
Evaluation of model performance of the individual questionnaires
Variable sets assessed were A) VPSQ, Vulnerable Personality Style Questionnaire B) RS, Resilience Scale C) ASQ, Attachment Style Questionnaire, and D) SOC, Sense of Coherence scale-29. The machine learning models tested are Ridge regression, LASSO regression, Gradient boosted machines, Distributed random forest, Extremely randomized trees (XRT), Naïve Bayes, and Stacked Ensemble. Models were assessed for accuracy (ACC), sensitivity (SENS), specificity (SPEC), positive predictive value (PPV), negative predictive value (NPV), and area under the curve (AUC). The bars represent the level of performance measures (in percent) and the table below the bar plot enumerate the levels. The whiskers represent 5% variation in the data values.

A)

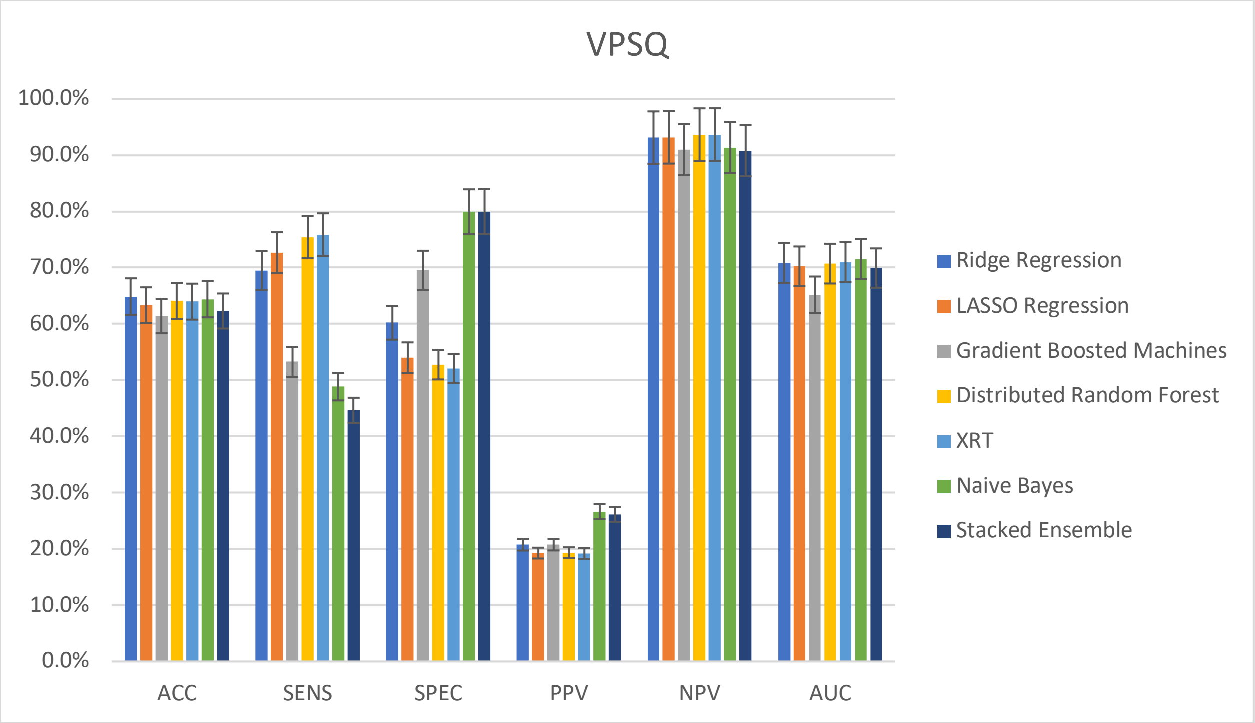

VPSQ
100.0%
90.0%
80.0%
70.0%
60.0%
50.0%
40.0%
30.0%
20.0%
10.0%
0.0%
ACC
SENS
SPEC
PPV
NPV
AUC
Ridge Regression
LASSO Regression
Gradient Boosted Machines
Distributed Random Forest
XRT
Naive Bayes
Stacked Ensemble

B)

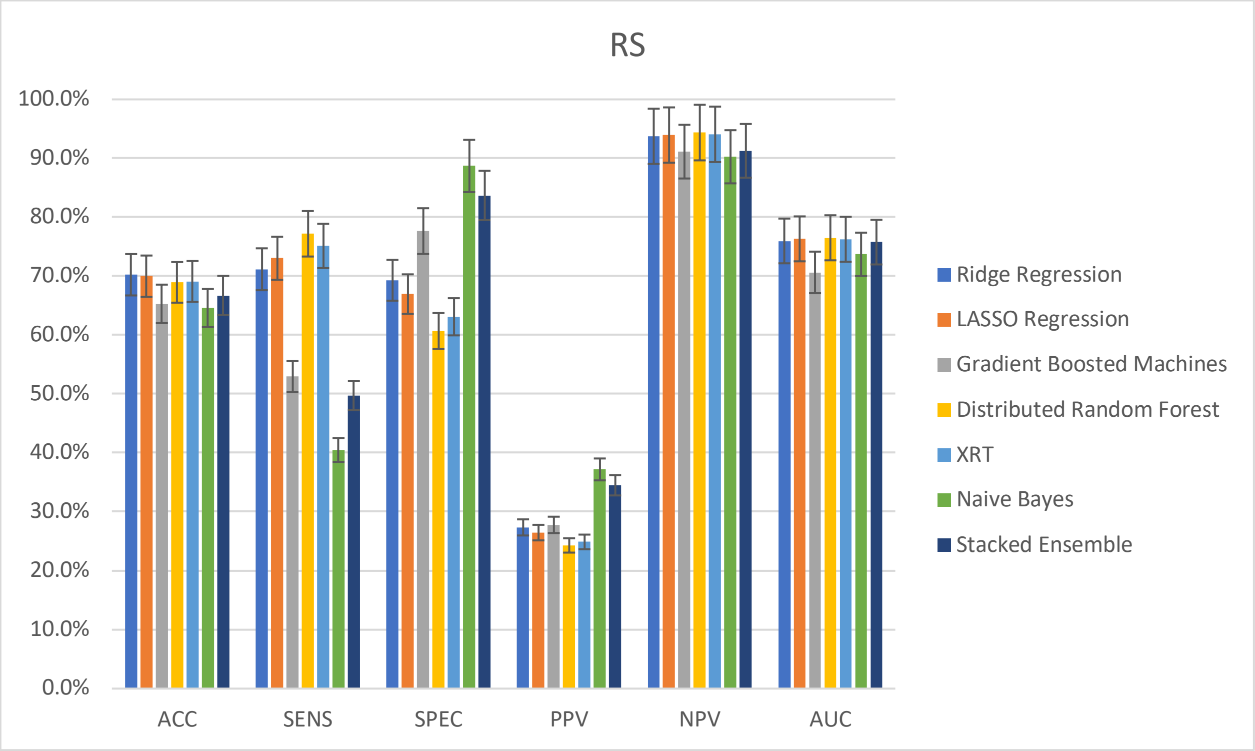

RS
100.0%
90.0%
80.0%
70.0%
60.0%
50.0%
40.0%
30.0%
20.0%
10.0%
0.0%
ACC
SENS
SPEC
PPV
NPV
AUC
Ridge Regression
LASSO Regression
Gradient Boosted Machines
Distributed Random Forest
XRT
Naive Bayes
Stacked Ensemble

C)

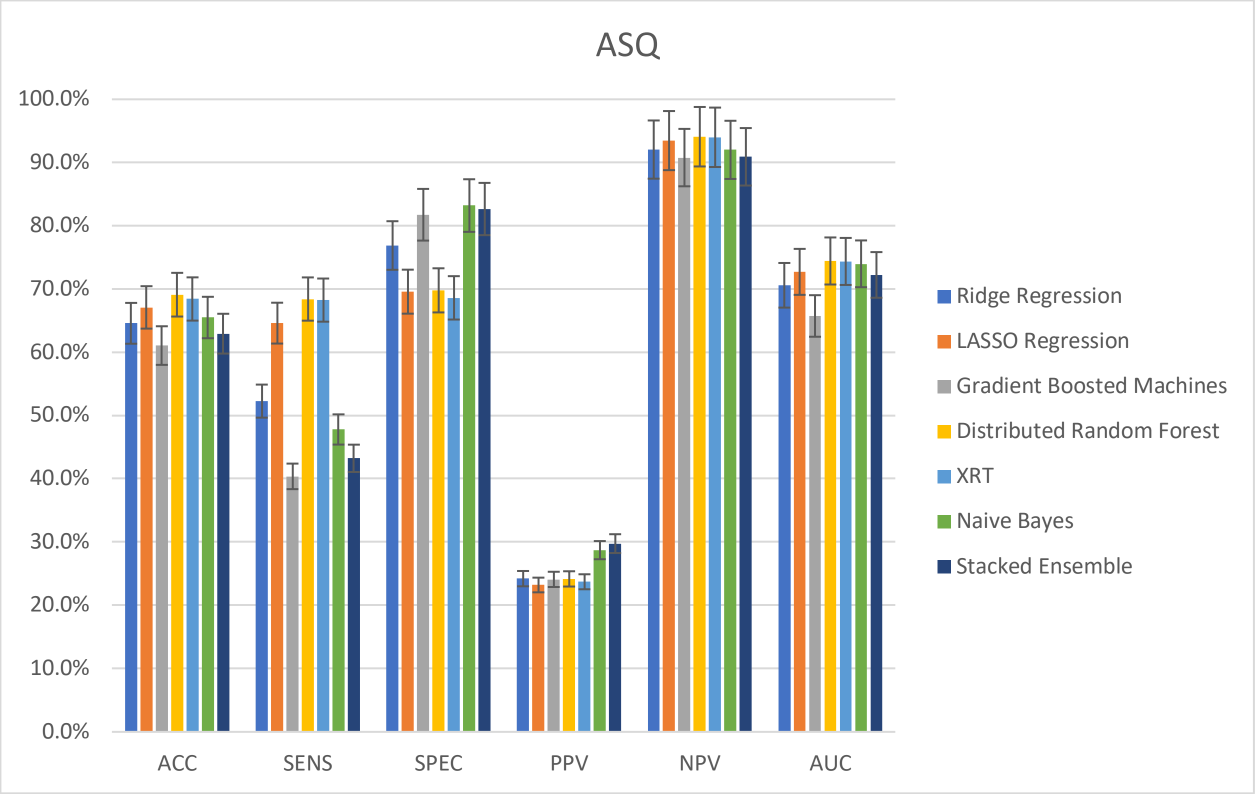
ASQ
100.0%
90.0%
80.0%
70.0%
60.0%
50.0%
40.0%
30.0%
20.0%
10.0%
0.0%
ACC
SENS
SPEC
PPV
NPV
AUC
Ridge Regression
LASSO Regression
Gradient Boosted Machines
Distributed Random Forest
XRT
Naive Bayes
Stacked Ensemble

D)

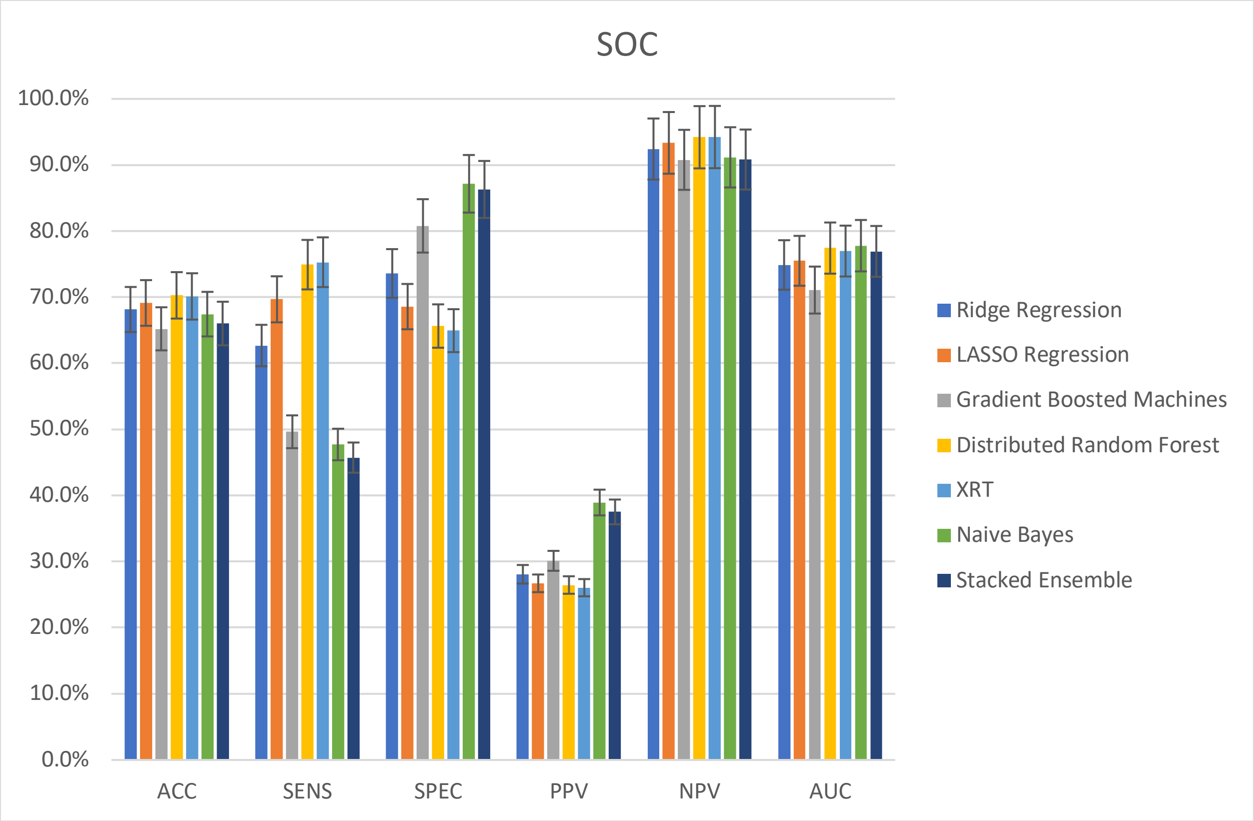

References

1 Antonovsky, A. The structure and properties of the sense of coherence scale. *Social Science & Medicine* **36**, 725-733, doi:10.1016/0277-9536(93)90033-Z (1993).

2 Aiena, B. J., Baczwaski, B. J., Schulenberg, S. E. & Buchanan, E. M. Measuring resilience with the RS–14: A tale of two samples. *Journal of Personality Assessment* **97**, 291-300 (2015).

3 Wagnild, G. M. & Young, H. M. Development and psychometric evaluation of the Resilience Scale. *J Nurs Meas* **1**, 165-178 (1993).

4 Feeney, J. A., Noller, P. & Hanrahan, M. Assessing adult attachment: Developments in the conceptualisation of security and insecurity', in attachment in adults: Clinical and developmental perspectives. (1994).

5 Boyce, P., Hickey, A., Gilchrist, J. & Talley, N. J. The development of a brief personality scale to measure vulnerability to postnatal depression. *Archives of Women's Mental Health* **3**, 147-153, doi:10.1007/s007370170012 (2001).

6 Gelabert, E. *et al.* The vulnerable personality style questionnaire: psychometric properties in Spanish postpartum women. *Archives of women's mental health* **14**, 115-124 (2011).
